# Limited nucleotide changes of HIV-1 subtype B Rev response element in China affect overall Rev-RRE activity and viral replication

**DOI:** 10.3389/fmicb.2022.1044676

**Published:** 2022-12-12

**Authors:** Yuting Shi, Jingwan Han, Bo Zhu, Zhi Liu, Qingmiao Liang, Chunlin Lan, Zhengyang Li, Hanping Li, Yongjian Liu, Lei Jia, Tianyi Li, Xiaolin Wang, Jingyun Li, Bohan Zhang, Junjun Jiang, Lin Li

**Affiliations:** ^1^Guangxi Key Laboratory of AIDS Prevention and Treatment, School of Public Health, Guangxi Medical University, Nanning, China; ^2^Department of AIDS Research, State Key Laboratory of Pathogen and Biosecurity, Beijing Institute of Microbiology and Epidemiology, Beijing, China; ^3^School of Graduate Studies, Guangxi Medical University, Nanning, China

**Keywords:** HIV-1, Rev response element, subtype B, functional activity, virus replication

## Abstract

The HIV-1 Rev response element (RRE) is a cis-acting RNA element that facilitates the nuclear export of mRNA-containing introns by binding specifically to the Rev protein, enabling a critical step in the viral replication cycle. This study aims to determine the subtype-specific loci of HIV-1 subtype B RRE circulating in China and to analyze their effects on Rev-RRE function and HIV-1 replication. We amplified 71 HIV-1 subtype B RRE full-length sequences from the HIV patients’ blood samples collected in China, analyzed the subtype-specific loci on them by comparing them with subtype B in the United States, and predicted their RNA secondary structures. Rev-RRE activity assay was used to test the binding activity of Rev and different RREs. Infectious clones were mutated to test the effect of the subtype-specific loci on replication capacity. In this study, two sites were determined to be the subtype-specific loci of HIV-1 subtype B RRE circulating in China. Both site 186 and site 56-57insAAC can significantly increase the viral mRNA transcription and Rev-RRE activity, but only the site 186 can significantly improve viral replication ability. Collectively, the subtype-specific loci of subtype B RRE circulating in China have a significant effect on the Rev-RRE activity and viral replication. This study investigates the subtype-specific loci of RRE, which are unique to retroviruses and essential for viral replication, and will help to explore the reasons why subtype B circulating in China is more widespread and persistent than American subtype B in China at the genetic level, and will provide theoretical support for the development of more inclusive detection and treatment methods for subtype B circulating in China. At the same time, it will also provide insight into the impact of different subtype HIV-1 genetic characteristics on viral replication.

## Introduction

Due to the tremendous genetic variability of HIV-1, there are many different subtypes and prevalent recombinant strains. Among them, subtype B strains were the first to be identified and are widely prevalent in Europe and the United States (US) (Barré-Sinoussi et al., 1983; Gilbert et al., 2007), which accounts for more than 90% of the HIV-1 epidemic in North America (Hemelaar et al., 2011). Additionally, among the main prevalent strains in China are CRF07_BC, CRF01_AE, CRF08_BC, and subtype B (Xu et al., 2013).

**Figure 1:**
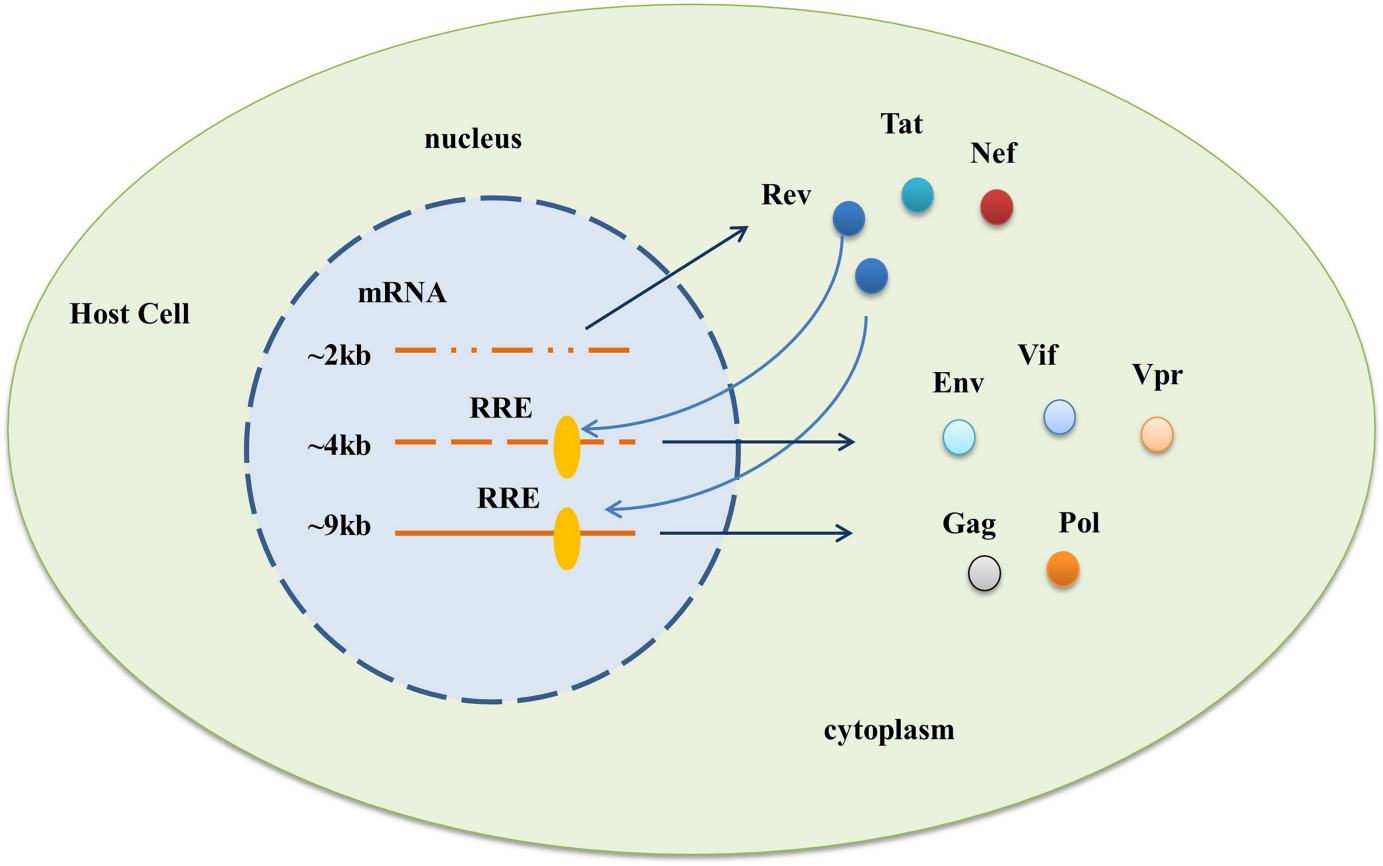
Functional requirement of the HIV-1 RRE. After HIV-1 enters the cell, three different mRNAs are produced through diverse editing, and the action of RRE allows the expression of viral proteins in two phases. In the early stage, only ~2 kb of fully edited transcripts can be directly transported out of the nucleus to express Rev, Tat, and Nef proteins, while partially edited ~4 kb and unedited ~9 kb transcripts containing the complete RRE sequence cannot be transported out of the nucleus. At a later stage of expression, the Rev protein reaches a threshold outside the nucleus, shuttles back to the nucleus, and brings both parts of the mRNA into the cytoplasm through binding with the RRE to express viral proteins such as Env, Vif, Vpr, and Gag, Pol, respectively.

HIV-1 RRE is a highly structured RNA sequence with a length of ~350 nt, which is a cis-acting RNA element directly acting in the form of RNA. It is located in the env coding region of the viral genome and exists in all viral mRNA-preserving introns (Fernandes et al., 2012; Wynn et al., 2016). It can bind to the Rev protein, an important regulatory protein of HIV-1, to facilitate the transport of viral mRNA out of the nucleus for expression and play a critical role in the process of HIV-1 replication (Daly et al., 1989; Malim et al., 1990; Jeang et al., 1991; Pollard and Malim, 1998). HIV-1 produces three major classes of mRNA through diversified editing. At the early stage of expression, only ~2 kb fully edited transcripts can be transported out of the nucleus to express the Rev protein through TAP/NXF1-dependent mRNA transport pathways commonly used by host cells, while unedited ~9 kb transcripts and incomplete ~4 kb transcripts remain in the nucleus temporarily (Taniguchi et al., 2014). The RRE sequences of the two transcripts were intact. At the late stage of expression, the Rev protein reaches a threshold outside the nucleus, shuttles back to the nucleus, and carries the viral mRNA into the cytoplasm by binding and polymerization to the RRE, enabling the viral protein to be expressed (Frankel and Young, 1998; Dai et al., 2019; Jackson et al., 2020). See Figure 1.

Early studies on the function of Rev-RRE mainly focused on the Rev protein. With an in-depth understanding of the function of Rev-RRE, people gradually realized that the sequence characteristics of RRE have a much greater impact on the activity of Rev-RRE than the Rev protein (Sloan et al., 2013). The cohort study of Phuphuakrat and Auewarakul (2005) proved that the diversity between RRE sequences of the same subtype has a significant impact on the functional activity of Rev-RRE and the pathogenicity of the virus. Besides, some have demonstrated that the change of a single base in RRE can also significantly change the functional activity of Rev-RRE and the formation of related complexes (Shuck-Lee et al., 2011). Sherpa et al. (2019) observed the activity differences between homologous pairs of Rev-RRE from different patients and different time points of the same patient and found that, compared with early time point RRE, late time point RRE only had 4 nucleotide changes but its functional activity was 2 to 3 times higher. Notably, the major Rev sequences present at early time points persist at late time points, suggesting that limited nucleotide changes in RRE are the primary driver of differential Rev-RRE activity (Sherpa et al., 2019). In addition, the activity of RRE changes with the time of infection, and it has been observed in long-term cohort studies that the rate of decrease of CD4 + T cells is related to the activity of RRE in the late stage of the disease, indicating that the RRE heterogeneity is an important regulatory factor of HIV pathogenesis and disease progression (Phuphuakrat and Auewarakul, 2005). Therefore, further study of the structural and functional evolution of the Rev-RRE system in natural infection is necessary to understand the role of this regulatory axis in the adaptation of HIV-1 to various immune environments, which may be conducive to the development of Rev-RRE-targeted therapy.

The HIV-1 subtype B circulating in China differs significantly from the subtype B in the United States in many aspects, most notably that the American subtype B is rare in China, while the subtype B now prevalent in China is clearly divided into 2 clusters in the evolutionary tree from the American subtype B. The widespread prevalence of this B subcluster in China is significantly associated with changes in transmission and replication capacity due to its genetic characteristics, in addition to the founder effect. This study investigates the subtype-specific loci of RRE, which are unique to retroviruses and essential for viral replication, and will help to explore the reasons why subtype B circulating in China is more widespread and persistent than American subtype B in China at the genetic level, and will provide theoretical support for the development of more inclusive detection and treatment methods for subtype B circulating in China. At the same time, it will also provide insight into the impact of different subtype HIV-1 genetic characteristics on viral replication.

In this study, 71 HIV-1-infected patients from China were collected and identified as subtype B through the Pol region, and the full-length RRE sequence was amplified for analysis. The effects of limited nucleotide changes in RRE on Rev-RRE functional activity and viral replication were analyzed by Rev-RRE function assay, replication kinetics, and qPCR experiments to explore the mechanism behind RRE heterogeneity affecting viral replication.

## Materials and methods

### Clinical samples and ethics statement

Inclusion criteria for the sample were confirmed HIV-1 antibody positivity, never received antiretroviral therapy (ART), and pol-zone subtype B; exclusion criteria were already on ART and pol-zone subtype non-B. Clinical samples were mainly from Guangdong, Henan, and Hebei provinces in China and were plasma samples. All subjects provided written informed consent.

### Viral RNA extraction and cDNA synthesis

Viral RNA was extracted from 1 mL of plasma using the guanidine extraction protocol (Palmer et al., 2003) as described previously. Viral cDNA synthesis was performed immediately after viral RNA extraction. Reverse transcription was performed at 50°C for 32 min to obtain the cDNA product, and then the cDNA product was subjected to PCR amplification to obtain the DNA product. PCR amplification was performed by adding 2 μL of cDNA template to 23 μL of reaction mixture containing high-fidelity polymerase (Takala) (Kearney et al., 2009). Primers for PCR were RRE7355-U (5′-TTGTRGAGGRGAATTTTTCTACTGTAA-3′) and RRE8262-D (5′-ACAGCCANTTTGTTATGTYAAACCA-3′). Amplicon size was verified by agarose gel electrophoresis.

### Single-genome sequencing and sequence analysis

The PCR products obtained by amplification were sequenced by single gene sequencing technology, and then the full-length RRE sequence was obtained by Contig software. The obtained sequences were compared and edited using BioEdit. The sequences were subtyped using BLAST, a genotyping tool available in the HIV nucleic acid Sequence Library of Los Alamos National Laboratory, United States.

We tested the model on the sequence data using Smart Model Selection (SMS) in the PhyML 3.0 program and calculated GTR + G + I as the optimal nucleotide substitution model (Lefort et al., 2017). Then, we selected this model and constructed the maximum likelihood tree using MEGA v6 (1,000 repetitions), and the final maximum likelihood tree was visualized using the program iTol v6.^1^ And the gene dispersion rate was calculated by MEGA v6. Download the HIV-1 subtype B standard strain in China and all RRE reference sequences (629) in the United States from the Los Alamos HIV gene database for RRE phylogenetic analysis [Main Search Interface of HIV Sequence Database (lanl.gov)]. Inclusion criteria for US HIV-1 subtype B RRE sequences were HIV-1 subtype B prevalent in the United States, sequences containing the full length of the RRE sequence, and one sequence for one patient; exclusion criteria were HIV-1 non-subtype B, sequences with missing RRE sequences, and duplicate sequences for the same patient.

### Construction of Gag/Pol-RRE reporter gene system

Rev-RRE functional activity was determined by the Gag/Pol-RRE reporter gene system, which consisted of the Gag/Pol-RRE reporter plasmid and the Rev expression plasmid. The Gag/Pol-RRE reporter plasmid was constructed by amplifying the Gag/Pol and RRE coding sequences of HIV-1 subtype B in the United States (NL4-3) and concatenating them together into the eukaryotic expression vector pcDNA3.1(+). Meanwhile, the coding sequences of the Rev protein of NL4-3 was amplified and inserted into the eukaryotic expression vector pcDNA3.1(+) to construct the Rev expression plasmid *in vitro*. Subsequently, the subtype B Gag/Pol-RRE reporter plasmid and Rev expression plasmid in China were generated by replacing the RRE and Rev regions of the Gag/Pol-RRE reporter gene system-NL4-3 with the RRE consensus and Rev consensus sequence (base frequency > 60%), respectively (Sherpa et al., 2015; Jackson et al., 2016). Primer information was shown in Table 1.

**Table 1:** Primer information for the Gag/Pol-RRE reporter gene system.

Primers	Primer sequence (5′-3′)	Direction
Gag/Pol-F	AGATCTCTCGACGCAGGACTCGGCT	Forward
Gag/Pol-R	CACCCAATTCTGAAATGGATAAACA	Reverse
RRE-F	GTAGCACCCACCAAGGCAAAGAGAA	Forward
RRE-R	TAGCATTCCAAGGCACAGCAGTGGT	Reverse
Rev 1-F	ATGGCAGGAAGAAGCGGAGACAGCG	Forward
Rev 1-R	TGCTTTGATAGAGAAGCTTGATGAG	Reverse
Rev 2-F	ACCCACCTCCCAATCCCGAGGGGAC	Forward
Rev 2-R	CTATTCTTTAGTTCCTGACTCCAAT	Reverse

### Rev-RRE activity assays

In this experiment, as mentioned above (Sloan et al., 2013), 1.0 × 10^5^ HeLa cells/well were inoculated into 12-well tissue culture plates in 1 mL cell culture medium 24 h before transfection. HeLa cells were transfected with 1 μg Gag/Pol-RRE reporter gene and 0–64 ng Rev expression plasmids *via* the lipofectamine 2000 transfection reagent. Cell supernatant was collected 48 h after transfection, and the p24 level was measured by ELISA, and the dose–response curve of Rev-RRE activity was plotted. The Rev-RRE activity was measured by quantification of p24 secreted into the cell culture medium, with relative activity measured as the average slope of the best fit line.

### Spreading infections

A total of 24 h before transfection, 1.2 × 10^6^ 293 T cells/well were inoculated into a 6-well tissue culture plate in 2 mL of cell culture medium. 4 μg of HIV-1 proviral DNA (NL4-3) was transfected into 293 T cells using Lipofectamine 2000 transfection reagent. Supernatants containing infectious virus particles were harvested 48 h after transfection, and p24 was measured by ELISA to quantify the amount of virus present, and these viral reservoirs were used to infect MT2 cells. In 6-well tissue culture plates, 4 × 10^5^ MT2 cells/well were cultured in a volume of 1 mL of the normal growth medium and then infected with infected with an equal amount of p24 (5 ng) of virus supernatant. 6 h later, the cell precipitate was gently washed with phosphate-buffered saline (PBS) to remove the unattached virus, centrifuged (350 g, 5 min), and transferred to 2 mL of a fresh normal medium in a 6-well plate. 300 μL of virus supernatant was taken at day 0 and every 2 days, respectively, and the corresponding volume of fresh culture medium was supplemented to keep the total culture volume at 2 mL. The amount of p24 in the supernatant at each time point was quantified by ELISA and used to map virus growth.

### p24 antigen analysis

The p24 antigen in the supernatant was quantified using the HIV-1 p24 antigen ELISA kit (Biomedical Engineering Center, Hebei Medical University, China) according to the manufacturer’s protocol (Li et al., 2021).

### Quantification of HIV-1 mRNA transcripts and virus replication in MT2 cells by quantitative PCR

We extracted total RNA using the RNeasy Plus Mini Kit (Qiagen NO. 74134) according to the manufacturer’s experimental protocol. All RNA samples were treated with 1 μL gDNA eraser per 1 μg of RNA (Takara, RR047A), to remove genomic DNA. We spiked treated samples or pure water controls with 10^2^ and 10^7^ DNA standards and compared their CT values, thinking to assess whether these gDNA eraser-treated samples had any residual DNAase activity or might inhibit PCR (Hurst et al., 2016). Subsequently, we reverse-transcribed the mRNA into cDNA using a mixed primer pair containing oligonucleotide dT primers (Takara, RR047A).

Real-time fluorescence quantitative PCR (qPCR) was performed with a Roche LightCycler 480 II System using TB Green® Premix Ex Taq™ (TaKaRa, Cat No. RR420A). Cycling conditions were a denaturation step at 95°C for 10 min, followed by 40 cycles of 10 s at 95°C, 5 s at 60°C, and 10 s at 72°C, followed by melting curve analysis to confirm the specificity of the PCR (Li et al., 2021). Absolute quantification of HIV-1 mRNA-RRE copies was based on available standard curves. RRE-F (5’-GCAGTGGGAATAGGAGCTTTGTTCC-3′) and RRE-R (5′- AGCCCTCAGCAAATTGTTCTGCTGC-3′) primers were used.

### Statistical analysis

SPSS 23.0 software was used for the statistical analysis of the data. Where appropriate, the measurement data met the normal distribution as described by (X ± S), and the hypothesis test between groups was adopted by the t-test or one-way ANOVA. All statistical analyses were performed based on a two-sided alpha of 0.05 (α = 0.05).

## Result

### Background information and demographic characteristics of the samples

Among 71 HIV-1-infected patients, 67.6% (48/71) were male, 54.9% (39/71) were over 41 years old, 57.8% (41/71) were heterosexual, and 31.0% (22/71) were homosexual. Guangdong province accounted for 69.0% (49/71), and Hebei province accounted for 15.5% (11/71). In this study, HIV/AIDS patients were mainly middle-aged and elderly men, and heterosexual transmission was the main way of transmission, mainly from Guangdong Province. See Table 2.

**Table 2:** Demographic and epidemiological characteristics of the patients.

Characteristic	Cases (N, %)
Age (year)	
≤20	1 (1.4)
21–40	31 (43.7)
≥41	39 (54.9)
Sex	
Male	48 (67.6)
Female	23 (32.4)
Route of infection	
Blood transmission	1 (1.4)
Intravenous drug use transmission	4 (5.6)
Homosexual transmission	22 (31.0)
Heterosexual transmission	41 (57.8)
Unknown	3 (4.2)
Region	
Hebei	11 (15.5)
Guangdong	49 (69.0)
Henan	11 (15.5)

### Subtype specificity of HIV-1 subtype B Rev response element circulating in China

**Figure 2:**
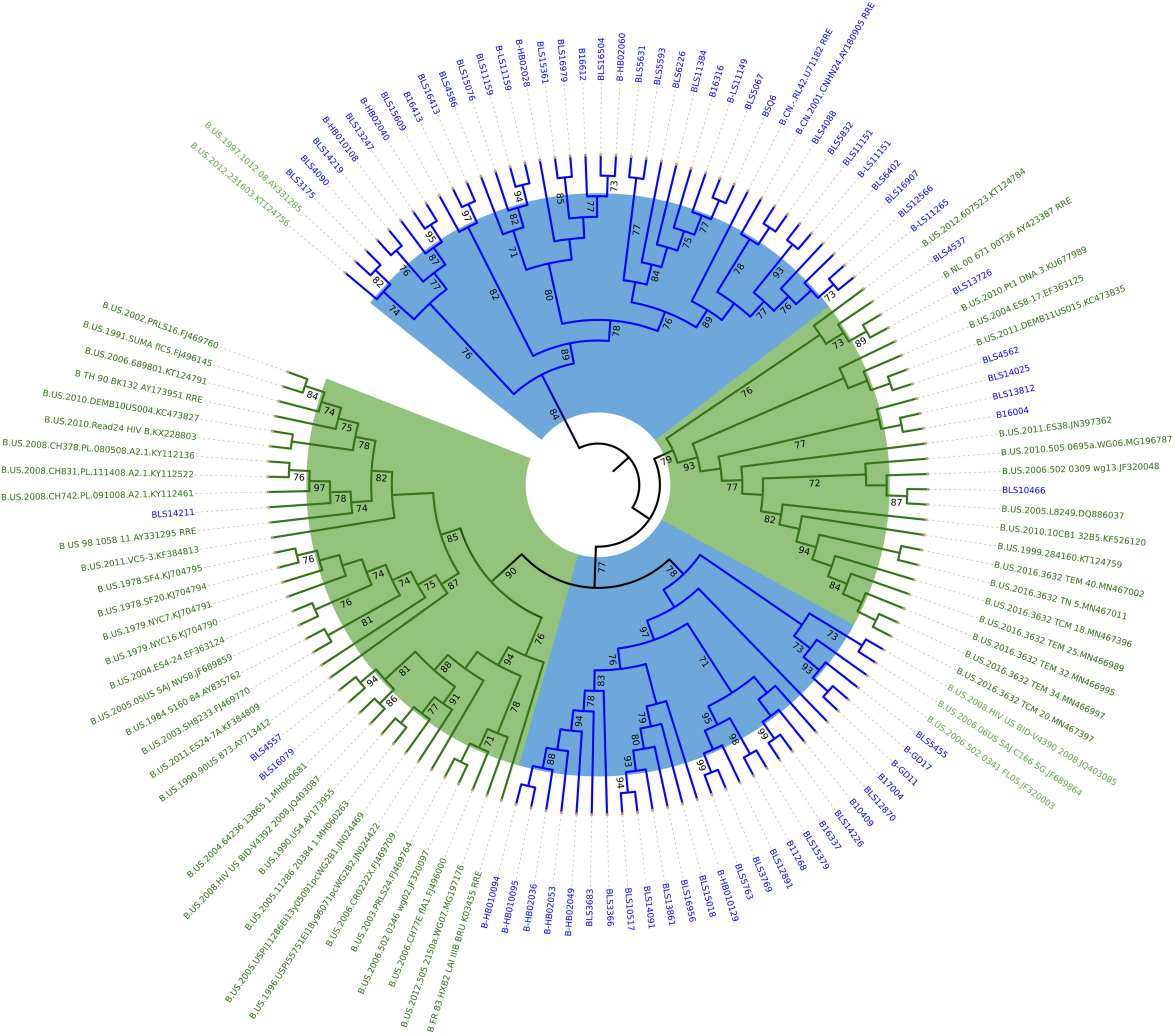
Phylogenetic analysis of HIV-1 subtype B RRE in China and the United States. Using the maximum likelihood method to construct the tree, the percentage of replicate trees in which the relevant taxa are clustered together in the bootstrap test (1,000 replicates) is shown next to the branches. The subtype B circulating in China is marked in blue and the American subtype B is marked in green.

We constructed a phylogenetic tree by combining the RRE sequences obtained by amplification in China with 2 standard strains of subtype B circulating in China (B.CN.2001.CNHN24.AY180905 and B.CN.RL42.U71182), 4 standard strains of subtype B in North America and Europe (B_FR_83_HXB2_LAI_IIIB_BRU_K03455, B_NL_00_671_00T36_AY423387, B_TH_90_BK132_AY173951, and B_US_98_1058_11_AY331295), and some subtype B in the United States downloaded from the United States Los Alamos HIV gene database. As seen from the phylogenetic tree, the 71 cases of HIV-1 subtype B RRE circulating in China sequences obtained by amplification clustered with the subtype B circulating in China standard strains, and the subtype B in the United States is clustered with its standard strain. See Figure 2.

### Analysis of subtype-specific loci for Rev response element

MEGA 6 software was used to calculate the gene dispersion rate of the RRE region of HIV-1 subtype B in China and the United States. The results showed that the gene dispersion rate of subtype B in China was (0.062 ± 0.023), and that in the United States was (0.056 ± 0.014). t-test showed that the difference in gene distance between the two subtypes was statistically significant (*p* < 0.001). See Table 3.

**Table 3:** Gene distances of two subtypes of HIV-1 RRE.

HIV-1 subtypes	Cases (N)	Genetic distance (x ± s)	*t* Value	*p* Value
Subtype B in the United States	629	0.056 ± 0.014	23.861	<0.001
Subtype B in China	71	0.062 ± 0.023		

In this study, 2 subtype-specific loci in the RRE sequences between HIV-1 subtype B in China and the United States were included, by calculating the base frequencies of both isoforms, sites with base frequencies >60% (site 186), and insertions or deletions (site 56-57insAAC). Among them, the mutation 186 site (G7895A) seems to be of particular interest because it is located in the critical structural domain between 163 and 221 of the RRE sequence, which is the key region where the RRE undergoes adaptive structural changes in the exercise of its function and is essential for maintaining the structure and function of the RRE (Ball et al., 2003). See Table 4.

**Table 4:** Analysis and statistics of subtype specific loci of RRE.

Mutation	Sites	Location in RRE	Nucleotide in HXB2	Base frequency (%)^a^		Reason to be selected
				China	United States	
Mut1	56-57insAAC	56–57	7,765 ~ 7,766	A (70.42) A (71.83) C (74.65)	~(64.39) ~(74.88) ~(74.56)	Insert
Mut2	186	Stem IV	7,895	A (61.97)	G (79.01)	Belong to 163–221^b^

a Percentage refers to the frequency of this base at this site of the subtype.

b The sequence of bits 163–221 of the RRE has been shown to be important in maintaining the structure and function of the RRE.

### Structural prediction of Rev response elements containing single-nucleotide mutations

The function of the HIV-1 RNA genome is determined by its sequence and its ability to fold back on itself to form a specific higher-order structure, and the replication and pathogenesis of the virus is closely linked to the structure of its RNA genome (Frankel and Young, 1998). Some investigators invented the high-throughput SHAPE (selective 2′-hydroxyacylation by primer extension analysis) technique, which uses many of the same tools as DNA sequencing to quantify the flexibility of the RNA backbone at single-nucleotide resolution and from which robust structural information can be immediately obtained. Processed SHAPE reactivities reflected the relationship of nucleotide positions, and highly reactive nucleotides (red and orange bars) reported flexible positions in the RNA (Wilkinson et al., 2008). Studies have reported alternative structures containing four or five stem-loops (SL) based on the SHAPE (Watts et al., 2009; Rausch and Le Grice, 2015). It has been suggested that the RRE can regulate HIV-1 replication kinetics by adopting different conformations that alter the nature of the resulting complex and the rate of viral replication (Booth et al., 2014; Jayaraman et al., 2014), and the 5-SL RRE is more active than the 4-SL structures (Sherpa et al., 2015). On the other hand, the Shannon entropy can be used to assess the ability of the RRE to adopt alternative conformations. Shannon entropy is calculated based on the probability of each base pair appearing in all possible structures predicted by the RNA. In general, regions with highly stable RNA structures are characterized by low Shannon entropies. Conversely, regions with high Shannon entropy are likely to form alternative conformations.

**Figure 3:**
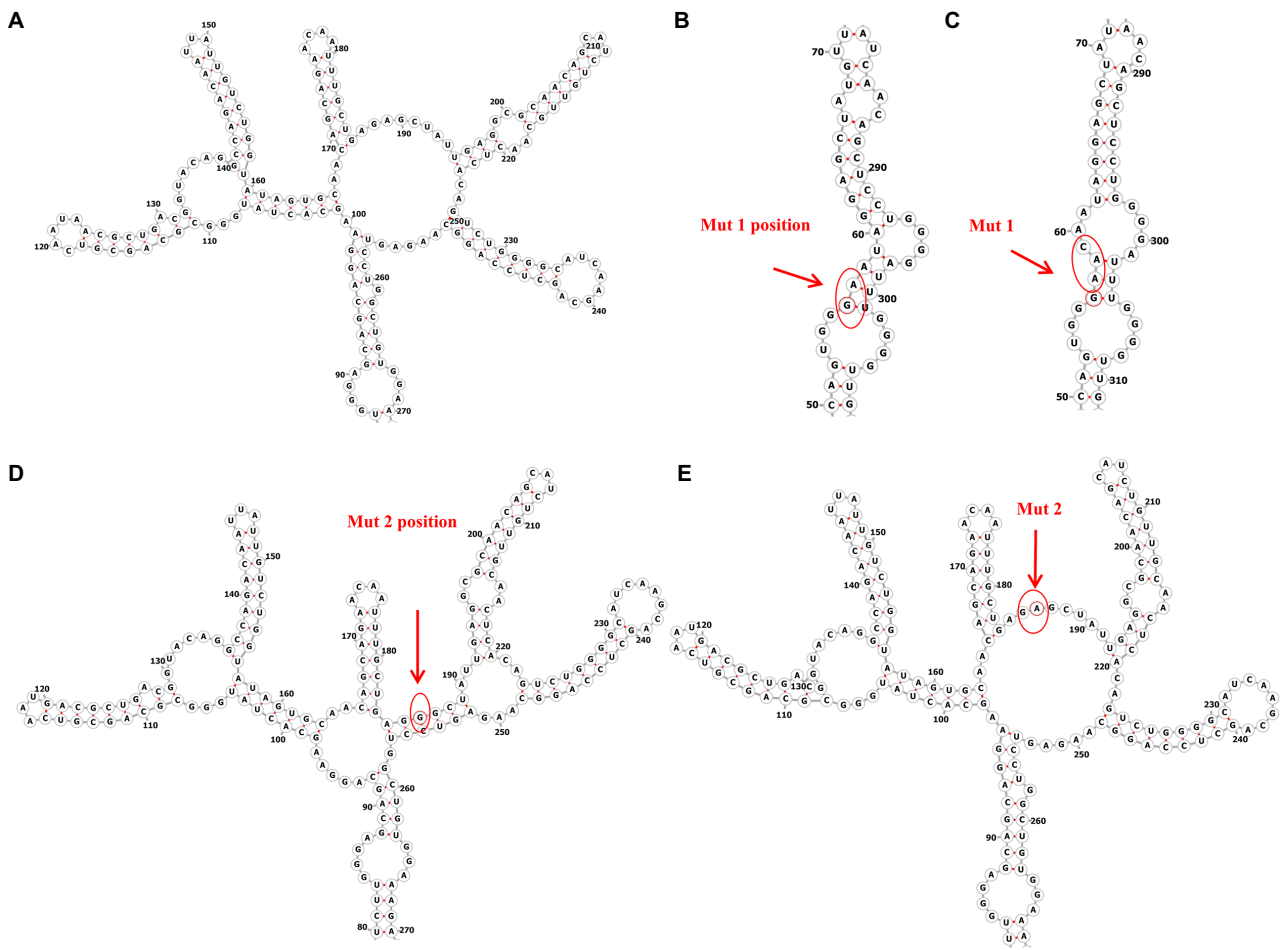
Predicted RNA secondary structure of subtype B RRE and each mutation site. **(A)** The RNA secondary structure of subtype B RRE circulating in China. **(B,D)** The RNA secondary structure of subtype B RRE in the United States. **(C,E)** Mutation at site RRE56-57ins AAC and site RRE-G186A accordingly.

**Figure 4:**
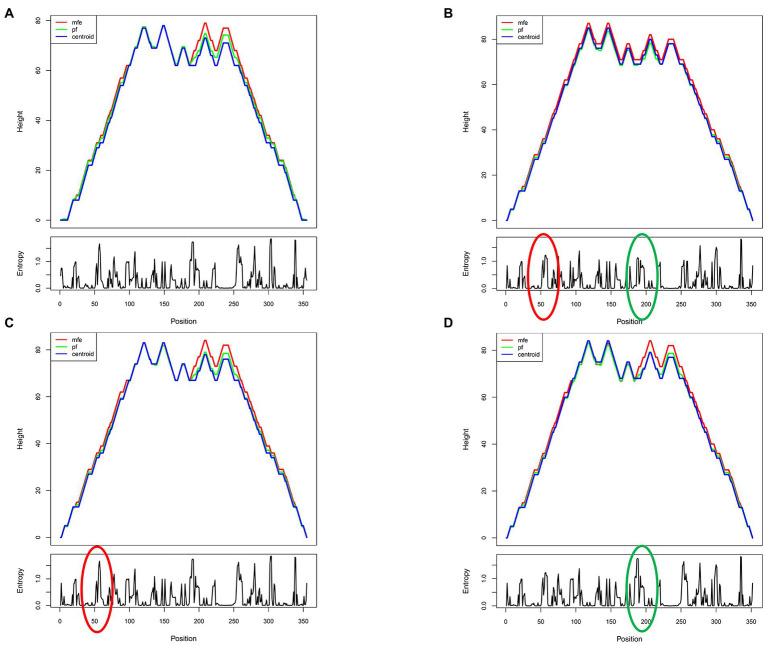
Predicted shannon entropy profile of subtype B RRE and each mutation site. **(A)** The Shannon entropy spectrum of subtype B in China. **(B)** The Shannon entropy spectrum of subtype B in the United States. **(C,D)** Mutation at site RRE56-57insAAC and site RRE-G186A, respectively.

Since the RRE structure appears to be the major contributor to the functional activity of Rev-RRE and regulation of viral replication, we next predicted the RNA secondary structure and calculated the Shannon entropy of HIV-1 subtype B RRE in China and the United States at the RNA secondary structure prediction website (RNAfold web server (univie.ac.at)). Meanwhile, the RNA secondary structure of the 186 locus and the 56-57insAAC locus mutant was predicted based on the RNA secondary structure of the American subtype B RRE. We show a mountain plot representation of the Minimum Free Energy (MFE) structure, the thermodynamic ensemble of RNA structures, and the centroid structure in Figures 3, 4. Additionally, we present the positional entropy for each position. The prediction results (Figures 3B–E) showed that the 186 locus mutant was a typical 5-SL structure compared to the RRE secondary structure of the subtype B in the United States, while the 56-57insAAC locus mutant was not significantly changed. Meanwhile, the predicted results showed that both mutant loci could increase the Shannon entropy value (Figures 4C,D).

### Analysis of the influence of Rev response element subtype characteristics on the functional activity of Rev-RRE

**Figure 5:**
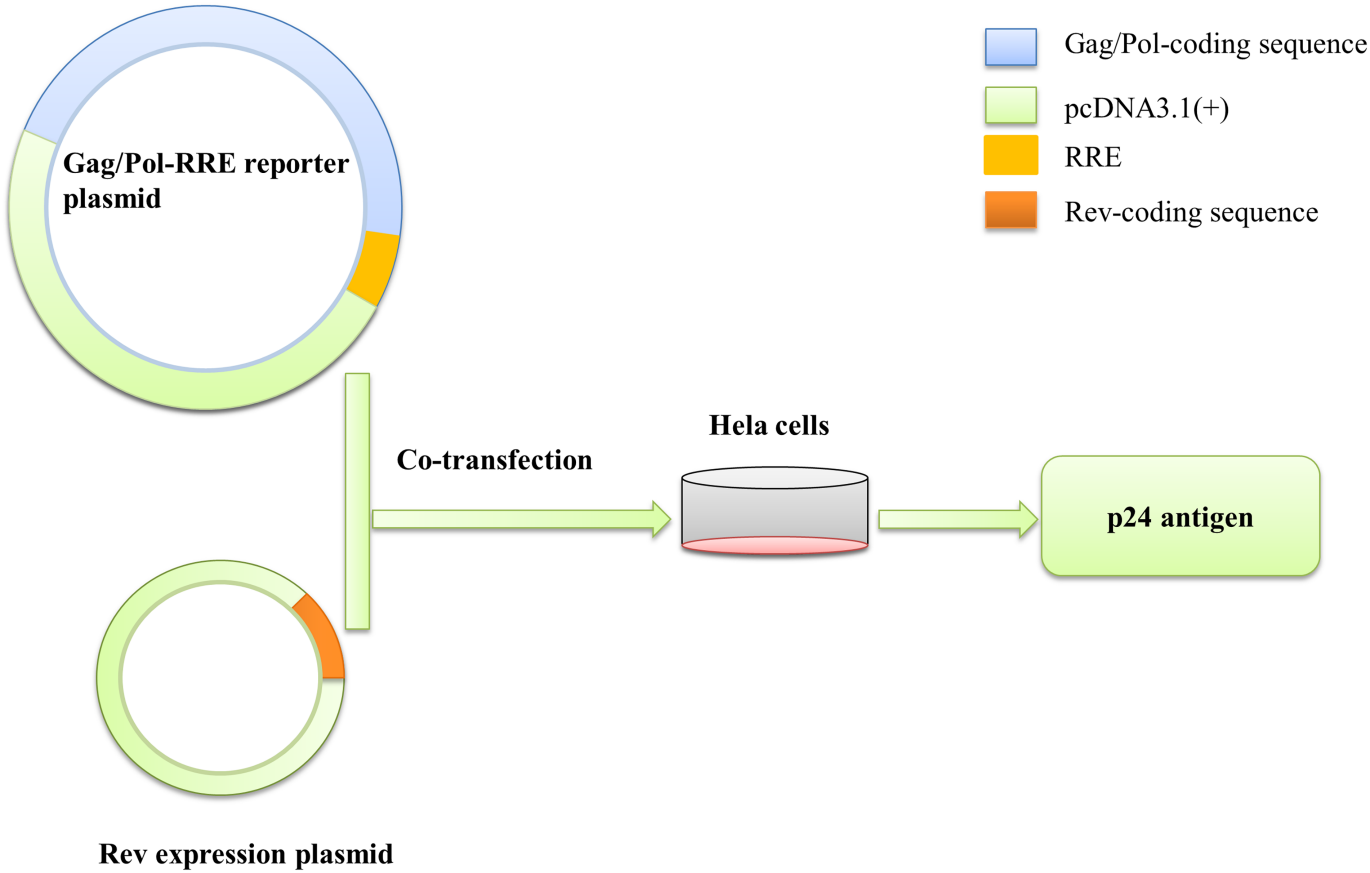
Gag/Pol-RRE reporter gene system to assess Rev-RRE functional activity. The Gag/Pol and RRE coding sequences were sequentially linked into the pcDNA3.1(+) vector to construct the Gag/Pol-RRE reporter gene expression plasmid, and the Rev coding sequence was linked into the pcDNA3.1(+) vector to construct the Rev expression plasmid *in vitro*. The two plasmids were co-transfected into HeLa cells, and the Rev-RRE functional activity was assessed by measuring the expression level of p24 antigen in the cell culture supernatant.

**Figure 6:**
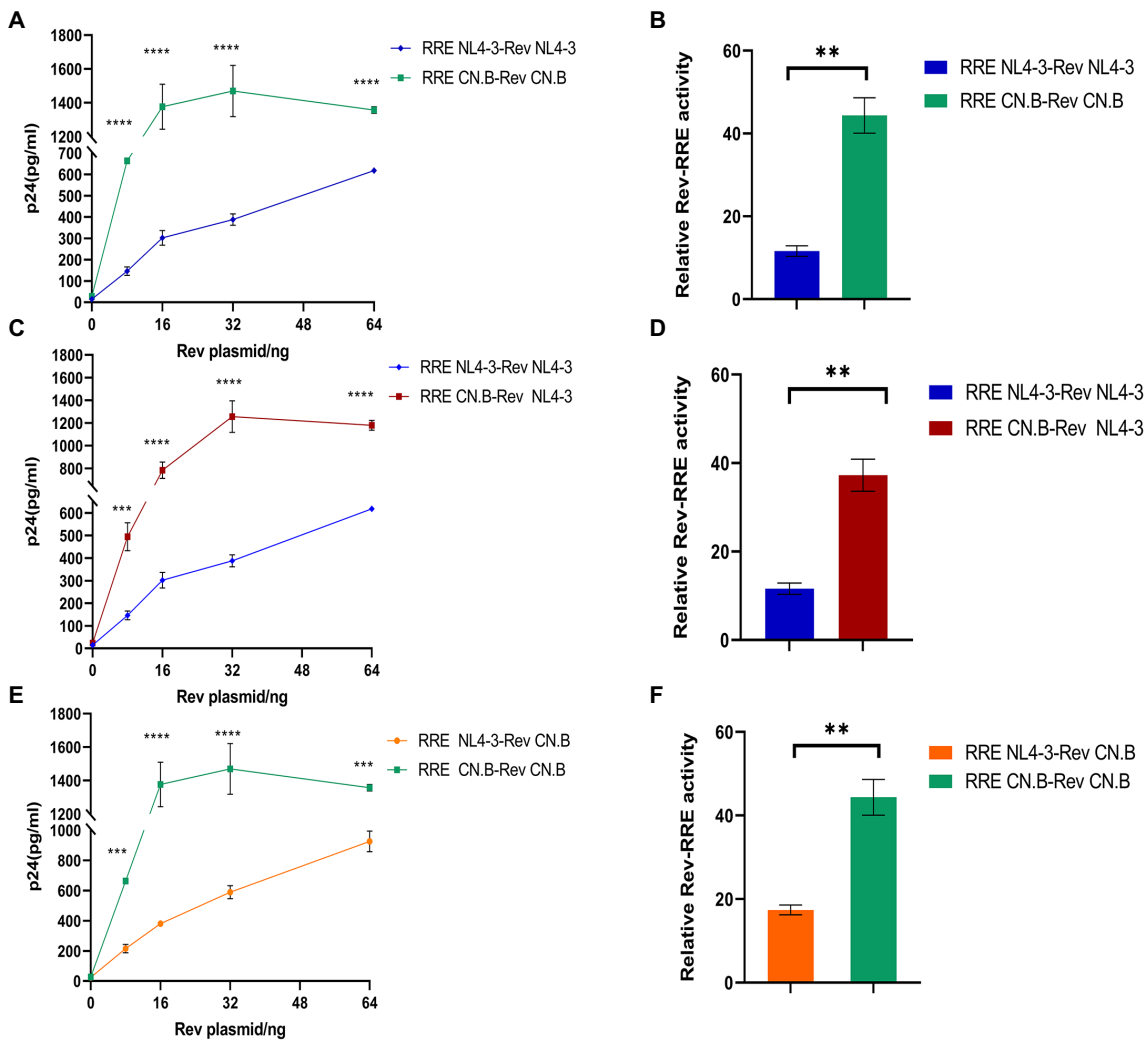
Influence of RRE subtype characteristics on functional activity of Rev-RRE. **(A,C,E)** The concentrations of p24 expressed by the reporter gene changed with the changes of Rev expression plasmid concentration when the two subtypes Rev were combined with the two subtypes RRE, respectively. **(B,D,F)** The difference in relative activity of Rev combined with RRE under corresponding conditions. Each experiment was repeated twice, and bars show the standard error of the mean from two replicates. *p < 0.05, **p < 0.01, ***p < 0.001, and ****p < 0.0001.

To test the functional activity of Rev-RRE, we used a transient transfection assay using HIV-1 Gag/Pol reporter plasmids in which Gag-Pol expression is entirely dependent on functional Rev and RRE (Figure 5). American subtype B (NL4-3) and subtype B in China (CN.B) Gag/Pol-RRE reporter plasmids were transfected into HeLa cells with an increase in the number of plasmids expressing the appropriate homologous Rev protein. The results (Figures 6A–F) showed that the relative functional activity sequence of different Rev-RREs was RRE CN.B-Rev CN.B > RRE CN.B-Rev NL4-3 > RRE NL4-3-Rev CN.B > RRE NL4-3-Rev NL4-3. Obviously, the relative activity of Rev-RRE containing subtype B RRE circulating in China was always higher than that containing NL4-3 RRE, regardless of which Rev protein was bound to it, and the difference was statistically significant (all *p* < 0.01), indicating that the functional activity of subtype B RRE circulating in China is greater than that of the United States, and that the functional activity of Rev-RRE is more affected by the subtype characteristics of RRE than the subtype specificity of Rev.

### Analysis of single-nucleotide mutations affecting the ability of the Rev response element to mediate HIV Gag/Pol expression

**Figure 7:**
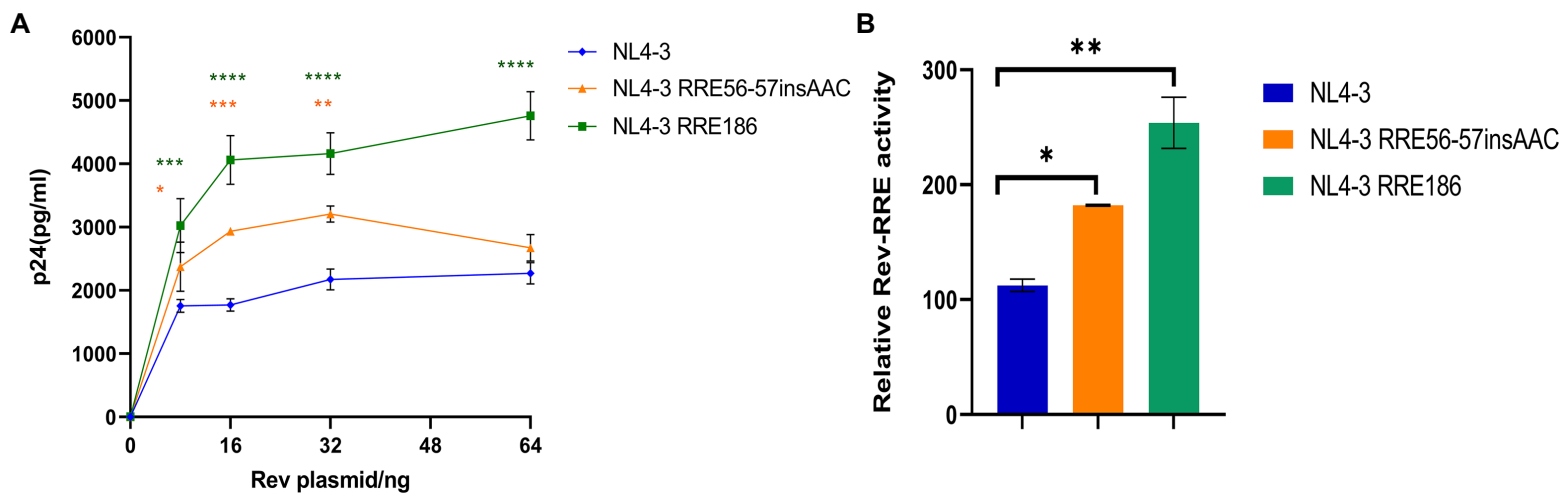
Relative function of NL4-3 and mutant RREs. **(A)** Gag/Pol reporter assay and variation of the concentration of p24 expressed by the reporter gene with the change of the concentration of the Rev expressing plasmid when NL4-3 and each mutant RRE were combined with NL4-3-Rev. **(B)** The relative activity of Rev-RRE when NL4-3 and each mutant RRE is combined with NL4-3-Rev. Each experiment was repeated twice, and bars show the standard error of the mean from two replicates. *p < 0.05, **p < 0.01, ***p < 0.001, and ****p < 0.0001, compared to NL4-3.

It has been shown that the structure and activity of the RRE are sensitive to minimal nucleotide variation (Shuck-Lee et al., 2011). Therefore, it is of interest to analyze the activity of RREs from different patients to determine the role of RRE sequence variability in mediating the differences in activity between different Rev-RREs. For this purpose, RRE mutant plasmids are generated by point mutation in the RRE region of the Gag/Pol-RRE reporter plasmid-NL4-3, and each RRE in the Gag/Pol-RRE reporter system was tested for activity using the same NL4-3 Rev. Then, each Gag/Pol-RRE reporter plasmid was co-transfected into HeLa cells with varying amounts of NL4-3 Rev-expressing plasmids. These results of this experiment (Figures 7A,B) show that each RRE variant promoted different levels of Gag/Pol expression in response to the concentration of the Rev plasmid used. Specifically, the following hierarchy of activity was observed in the RRE variants: RRE-G186A > RRE-56-57insAAC > NL4-3.

### Effects of single-nucleotide mutations in Rev response element on HIV-1 replication ability *in vitro*

**Figure 8:**
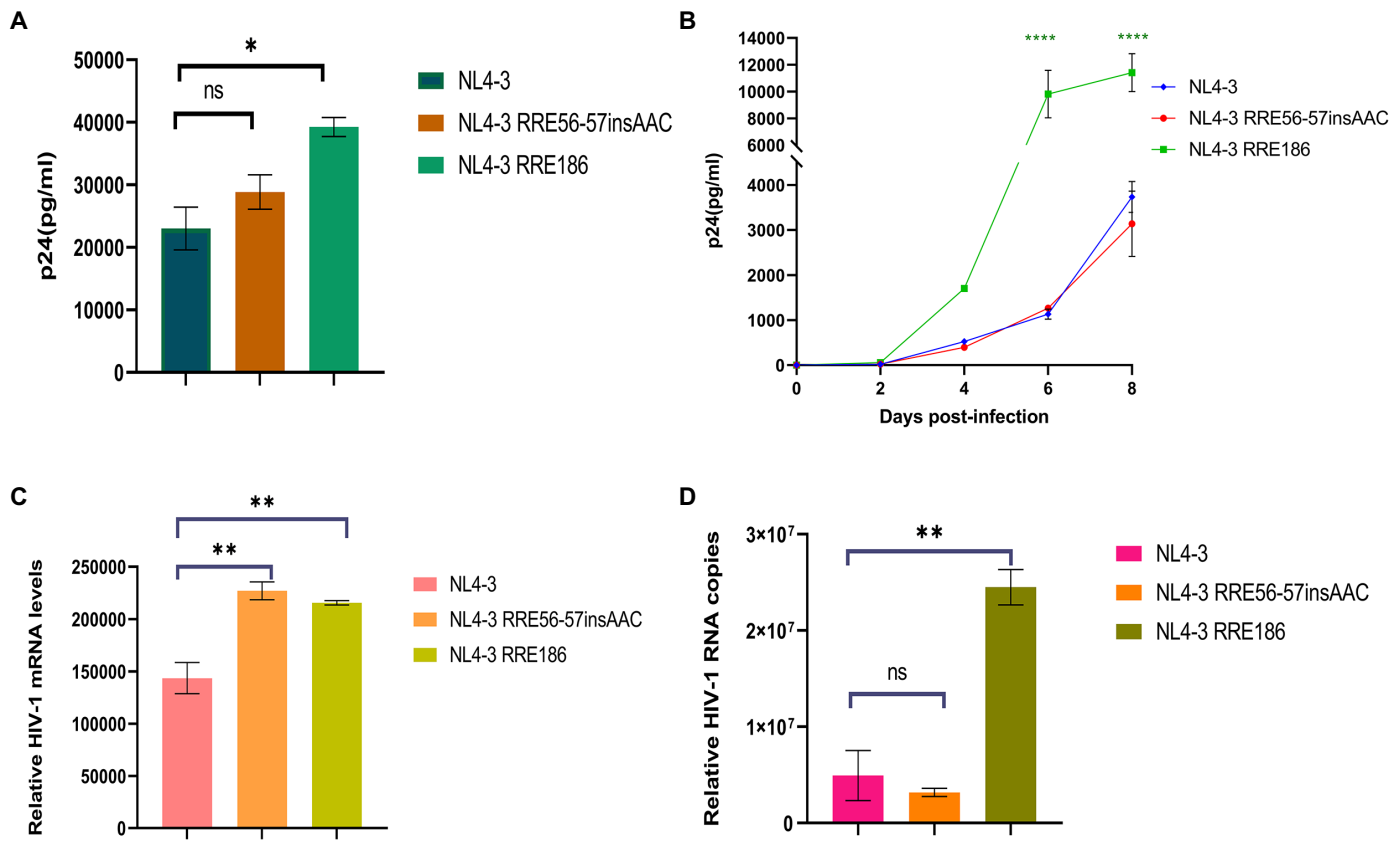
Relative viral replication levels of NL4-3 strain and mutant RREs. **(A)** The replication level of the HIV-1 virus after NL4-3 and mutant RREs plasmid transfection into 293 T cells. **(B)** The replication level of the corresponding virulent strain in MT2 cells. **(C)** The mRNA transcript levels in intracellular. **(D)** The viral RNA copies in cell culture supernatant. Each experiment was repeated twice, and bars show the standard error of the mean from two replicates. *p < 0.05, **p < 0.01, ***p < 0.001, and ****p < 0.0001, compared to NL4-3.

We used the NL4-3 infectious clone as a template, introduced the subtype characteristic sites into the NL4-3 infectious clone, constructed the corresponding mutant strain, and then transfected them into 293 T cells. After 48 h of transfection, the transfection supernatant was collected and the expression level of p24 was measured (Figure 8A). Subsequently, transfected supernatant with the same amount of p24 was used to spread infection in MT2 cells, and replication kinetics curves were plotted to analyze the effect of mutation sites on viral replication capacity (Figure 8B). The results of these experiments (Figures 8A,B) show that the replication capacity of infectious clone mutants corresponding to RRE-G186A was significantly increased compared with the NL4-3 strain (*p* < 0.05 in 293 T cells, *p* < 0.0001 in MT2 cells), while RRE-56-57insAAC had no statistical difference compared with that before mutation (*p* > 0.05).

MT2 cells were infected with the NL4-3 strain and the RRE mutant strain with the same amount of MOI (MOI = 0.1). After 48 h, culture supernatant and cells were collected, and RNA was extracted, respectively. The expression of mRNA in intracellular and viral RNA copies in culture supernatant was detected by qPCR, respectively (Figures 8C,D), and the results showed that RRE-G186A significantly increased the level of mRNA transcription and viral replication compared to NL4-3 (*p* < 0.01). Intriguingly, RRE-56-57insAAC significantly increased the intracellular mRNA transcript levels (*p* < 0.01), while there was no statistical difference in the virus replication (*p* > 0.05).

## Discussion

The high variability of HIV-1 makes it produce numerous subtypes with relatively independent gene sequence characteristics in the process of transmission, and form certain regional distribution characteristics in the global epidemic so that we can trace the source of infection according to its characteristics and study its variation rules. In this study, RRE genotyping combined with phylogenetic tree analysis showed that the samples of HIV-1 subtype B in China were approximately close to the standard strains B.CN.2001.CNHN24.AY180905 in Henan Province and B.CN.RL42.U71182 in Yunnan Province, indicating that the differences between these RRE sequences are minor and homologous. The greater the gene distance, the higher the degree of variation, indicating the earlier the appearance of this subtype and the longer the prevalence. The RRE sequences of subtype B in China and those of the United States have a large in group gene dispersion rate and low homology, and there is a significant difference in the gene dispersion rate between the two subtypes, suggesting that the RRE sequences of subtype B in China were significantly different from those of the United States, with obvious subtype specificity.

Some studies have suggested a potential link between Rev-RRE function and the pathogenesis of HIV-1. Early studies on the function of Rev-RRE mainly focused on the Rev protein. With an in-depth understanding of the function of Rev-RRE, people gradually realize that the sequence characteristics of RRE have a considerably greater impact on the activity of Rev-RRE than Rev (Sloan et al., 2013), which is also confirmed in this study. Clinical studies have confirmed that RRE with different activities can evolve over time (Phuphuakrat and Auewarakul, 2003). It has also been observed in long-term cohort studies that the rate of CD4 + T cell depletion is related to the activity of RRE in advanced diseases (Phuphuakrat and Auewarakul, 2005). Therefore, the evolution of RRE may be an essential regulatory factor for HIV-1 pathogenesis and disease progression.

This study also provides additional support for the hypothesis that the Rev-RRE regulatory axis plays an influential role in the pathogenesis of HIV-1. Early on, a cohort study proved that the diversity between RRE sequences of the same subtype has a significant impact on the functional activity of Rev-RRE and the pathogenicity of the virus (Phuphuakrat and Auewarakul, 2005). Subsequently, studies have confirmed that the shift of a single base on RRE can also significantly alter the functional activity of Rev-RRE and the formation of related complexes (Sloan et al., 2013). The two RRE mutants we identified in this study are well worthy of our discussion. Mutation RRE-G186A was able to significantly increase not only the expression level of mRNA in intracellular but also the functional activity of Rev-RRE and the replication ability of the virus, suggesting that this site may play a key role in the entire replication cycle of HIV-1 subtype B. Notably, mutation RRE-56-57insAAC was able to significantly increase the expression level of intracellular mRNA, as well as significantly increase the functional activity of Rev-RRE. However, there was no significant effect on the replication levels of the virus. These results suggest that this site may increase the transcription level of viral mRNA and enhance the ability of Rev-RRE-mediated mRNA translocation out of the nucleus, but fails to significantly affect the final replication of the virus, the mechanism behind which deserves further exploration. There are also limitations in this study, and further measurements of the relative amounts of nuclear and cytoplasmic RNA of different lengths would help to strengthen the analysis of the nuclear export capacity of each mutant RNA.

The RRE is a 4 or 5-SL structure that can influence the nature of the complex and regulate the rate of viral replication by changing conformation, and the 5-SL structure is more active in promoting viral replication than its 4-SL structure (Sherpa et al., 2015). Notably, limited nucleotide changes in the RRE have the potential to alter its RNA secondary structure and thus regulate the rate of viral replication. Sherpa et al. (2015) demonstrated that the secondary structure of the subtype B RRE at positions 163–221 of the nucleotide sequence composed of secondary structure is essential for the function of the Rev-RRE (Sherpa et al., 2015). In this study, we constructed RRE-G186A (site 186, G-to-A) that induced a 5-SL structure associated with increased activity, which significantly increased the functional activity of Rev-RRE and the replication efficiency of the virus. The data we obtained from this study support the idea that the ability of RRE to adopt different structural conformations that promote different replication activities may allow HIV-1 to modulate its replication rate under different conditions to better survive in the host environment. In addition, both RRE-56-57insAAC and RRE-G186A have some degree of increased Shannon entropy, suggesting that they are structurally dynamic, a feature that confers accessibility to protein-RNA interactions by acting as a landing pad for protein cofactors (Smola et al., 2016). This accessibility may contribute to Rev-RRE binding and subsequent multimerization, which leads to enhanced activity. This may explain why these two RRE mutants have higher Rev-RRE functional activity. While these studies suggest that alterations in RRE RNA conformation may lead to the formation of different Rev/RRE complexes and support different levels of functional activity, further structural studies will be required to understand in detail how 4-SL and 5-SL RREs contribute to different viral replication abilities.

In conclusion, limited nucleotide changes in the subtype B RRE circulating in China can significantly affect Rev-RRE activity and viral replication. Since Rev-RRE binding is a crucial step of viral replication, a clear understanding of RRE evolution will help to realize the possible role of this regulator in key aspects of the viral life cycle, which is also a promising target for drug development.

## Data availability statement

Sequences have been uploaded to GenBank (accession numbers: OP512693-OP512763). All data reported in this paper will be shared by the lead contact on request, LL (dearwood@sina.com).

## Ethics statement

The studies involving human participants were reviewed and approved by the ethics committees of the Beijing Institute of Microbiology and Epidemiology. The patients/participants provided their written informed consent to participate in this study.

## Author contributions

LL and JJ designed the study; HL, YL, LJ, TL, and XW collected sample and the demographic data; BZha, ZhiL, CL, and ZheL performed nucleic acid extraction and amplification; YS, JH, and BZhu performed plasmid construction, functional activity experiments, replication kinetics experiments, qPCR and other experiments. YS, QL, and JL performed sequence analysis and statistical analysis; YS, JH, JJ, and LL participated in the writing process. All authors contributed to the article and approved the submitted version.

## Funding

The study was supported by the National Natural Science Foundation of China (NSFC, 31900157 and 3180010279) and State Key Laboratory of Pathogen and Biosecurity (AMMS).

## Conflict of interest

The authors declare that the research was conducted in the absence of any commercial or financial relationships that could be construed as a potential conflict of interest.

## Publisher’s note

All claims expressed in this article are solely those of the authors and do not necessarily represent those of their affiliated organizations, or those of the publisher, the editors and the reviewers. Any product that may be evaluated in this article, or claim that may be made by its manufacturer, is not guaranteed or endorsed by the publisher.

## Footnotes

^1^ https://itol.embl.de/
